# Lessons from writing sessions: a school-based randomized trial with adolescent orphans in Rwanda

**DOI:** 10.3402/ejpt.v5.24917

**Published:** 2014-12-22

**Authors:** Johanna Unterhitzenberger, Rita Rosner

**Affiliations:** Clinical and Biological Psychology, Catholic University Eichstätt-Ingolstadt, Eichstätt, Germany

**Keywords:** Bereavement, prolonged grief, adolescents, orphans, school-based, disclosure, writing

## Abstract

**Background:**

Treatments for adolescents affected by long-term loss in low- and middle-income countries are lacking. As school-based interventions are cost-efficient and easy to disseminate, an evaluation of this treatment setting for adolescents is worthwhile.

**Objective:**

Examining the effect of a school-based unstructured emotional writing intervention (sensu Pennebaker, group 1) about the loss of a parent to reduce adaptation problems to loss, compared to writing about a hobby (group 2), and non-writing (group 3).

**Method:**

We randomly assigned 14–18-year-old Rwandan orphans to one of the three conditions (*n*=23 per condition). Before and after the intervention, subjects completed the Prolonged Grief Questionnaire for Adolescents and the Mini International Neuropsychiatric Interview for Children and Adolescents, Part A, on depression as self-report measures of long-term effects of early parental loss.

**Results:**

Repeated measures analyses of variance showed no differential effect for any of the three conditions but revealed a significant effect of time at posttest regarding grief severity. Reduction of grief symptoms was significantly higher in subjects with elevated grief. Depressive symptoms showed no significant change from pre- to posttest in the emotional writing condition, whereas they significantly decreased in the control condition.

**Conclusions:**

Results imply that unstructured, brief emotional writing might not be indicated in adolescents affected by early parental loss who show severe and long-term distress; a more structured approach seems recommendable.

Orphans, defined as children who have lost at least one parent (United Nations Children's Fund [UNICEF], 2008), run a high risk of developing mental health difficulties (Cluver & Gardner, 2007). Not only do they suffer from their losses, but they also face stigmatization and social isolation. In several African countries, psychosocial stress and psychiatric disorders were reported frequently in orphans (Makame, Ani, & McGregor, 2002; Nyamukapa et al., 2008; UNICEF, 2003).

It is estimated that, as a result of the genocide in Rwanda in 1994, approximately 300,000 children and adolescents were left orphaned (Des Forges, 1999). Major depression and posttraumatic stress disorder (PTSD) were found at high rates in this cohort in postwar Rwanda, ranging from 15.5 to 62% (Bolton, Neugebauer, & Ndogoni, 2002; Boris et al., 2008; Dyregrov, Gupta, Gjestad, & Mukanoheli, 2000; Neugebauer et al., 2009). It is official policy in Rwanda to keep the memory of the genocide alive in numerous ways. Among other things, there are two annual official holidays and a mourning week beginning on April 7, which marks the beginning of the genocide. During this time of remembrance, stories about the killings are told, TV shows report on the genocide, and schools organize memorial services and panel discussions. Hence, many adolescent survivors are aware of their parents’ violent deaths. Studies show that learning about traumatic deaths can be just as stressful for children as experiencing such events (Lieberman, Compton, Van Horn, & Ippen, 2003), and losing a loved one through traumatic or unexpected circumstances might increase the likelihood of developing prolonged grief disorder (PGD) (Kristensen, Weisæth, & Heir, 2012; Mannarino & Cohen, 2011). Therefore, we assumed that even 14 years after the genocide, adolescents and young adults in Rwanda represent a population with increased rates of traumatic stress, prolonged grief (PG), or depression.

Results on the trajectories of grief in parentally bereaved children and adolescents in the USA show that there is one group (10%) whose symptoms are persistent 33 months after the loss (Melhem et al., 2011), whereas the majority copes well over time. And although this sample can hardly be compared to Rwandan adolescents who 1) were very young when their parents died, and 2) were orphaned by murder, Schaal, Jacob, Dusingizemungu, and Elbert (2010) reported a rate of 7.3% for PG in Rwandan adult orphans 12 years after loss. As early loss is further associated with several mental health and social risks in childhood (Luecken, 2008), treatment might be indicated even years after bereavement as would be the assessment of grief symptoms.

Literature reviews emphasize the lack of traumatic stress research in low- and middle-income countries (LMICs) in general (Fodor et al., 2014), and of evidence-based interventions for children and adolescents in particular (Jordans, Tol, Komproe, & De Jong, 2009; Patel, Flisher, Nikapota, & Malhotra, 2008). Many studies in these areas are uncontrolled or show design weaknesses, and hardly any treatment effects have been found (Jordans et al., 2009). A popular setting for interventions with this group of patients in postwar countries and LMICs is school-based. A review of these programs also concludes that there is a scarcity of evaluation and evidence in this field, for example, RCTs, and PG as an outcome is scarcely assessed (Persson & Rousseau, 2009). Layne et al. (2001, 2008) reported reduced symptoms of PG in their school-based projects using a very structured, intense, and cognitive behavioral therapy (CBT-) focused approach.

Telling stories and writing narratives are seen as acceptable ways of reporting experiences in many cultures, and both are key elements of well-established interventions for PTSD and PG in children and adolescents (Cohen & Mannarino, 2004; Cohen, Mannarino, & Deblinger, 2006; Neuner et al., 2008). Writing is also one treatment strategy for PGD in adults (Rosner, Pfoh, & Kotoučová, 2011). Especially in Rwanda, telling stories has a high value in the society. Wherever you go, people will share their stories with you, fairy tales are discussed in school, and writing essays is scheduled in the curriculum. Neuner, Schauer, Klaschik, Karunakara, and Elbert (2004) elaborate on the oral culture in Africa and evaluated their Narrative Exposure Therapy with adults in Rwanda.

According to Pennebaker (1997), writing therapy is based on the theory of inhibition and disclosure which indicates that disclosing stress emotionally reduces stress and improves the person's well-being. The average effect sizes concerning mental health and general functioning after writing therapy remain small in meta-analyses (Frattaroli, 2006; Frisina, Borod, & Lepore, 2004; Smyth, 1998). Even though initial research found disclosure to be the superior condition regarding mood and affective symptoms (Lepore, 1997; Pàez, Velasco, & Gonzalez, 1999), there is evidence that—for example, for college students or participants after relationship break-up—writing about positive topics might be just as effective as emotional disclosure for measures of mood and number of doctor's visits (Burton & King, 2004; King, 2001; Lewandowski, 2009). Other studies showed no differences between either writing condition and a control condition with respect to well-being in children, college students, and adults (Baikie, Geerlings, & Wilhelm, 2012; Kloss & Lisman, 2002; Reynolds, Brewin, & Saxton, 2000).

Concerning bereavement-related writing, some studies showed significant positive effects for disclosure in adults (Lichtenthal & Cruess, 2010; Wagner, Knaevelsrud, & Maercker, 2006) and adolescents (Kalantari, Yule, Dyregrov, Neshatdoost, & Ahmadi, 2012). Hence, it seems to be a promising approach which can combine a school-based setting and culturally accepted story telling. However, there are also studies (with adult participants) that showed no effects at all (O'Connor, Nikoletti, Kristjanson, Loh, & Willcock, 2003; Range, Kovac, & Marion, 2000), or reported no significant differences in grief outcome between disclosure and control groups as all conditions improved (Bower, Kemeny, Taylor, & Fahey, 2003; Kovac & Range, 2002; Van der Houwen, Schut, Van den Bout, Stroebe, & Strobe, 2010; Stroebe, Stroebe, Schut, Van den Bout, & Zech, 2002). The inconsistent results stem from different methodological approaches (e.g., heterogeneous outcomes and measures) and diverse samples (e.g., adults and adolescents), but may also be related to different interventions. The three most effective writing interventions are more elaborated (e.g., CBT elements or individualized feedback) than the original writing approach such as Writing for Recovery (WfR; Yule et al., 2005), the only program for young people which was evaluated by Kalantari and colleagues.

We assumed that there are many unreported projects initiated by aid organizations using simple writing interventions in postconflict areas as a feasible way to offer psychosocial support. When those organizations are not affiliated with research, they usually do not evaluate their work empirically and we do not get the chance to reflect their results. Hence, an evaluation of the simple writing intervention in young people seemed necessary despite all progress in written disclosure research.

Summarizing the above we can state that 1) Rwandan orphans are likely to be a stressed sample in need of psychosocial interventions, 2) there are only a few studies reporting on interventions for children and adolescents from LMICs, 3) there is a scarcity of research on adaptation to loss in children and adolescents in those countries, and 4) although writing interventions are commonly used, research results are heterogeneous. We chose the initial Pennebaker approach for several reasons: Next to the possibility of helping many affected people simultaneously, within a short time, and in a low threshold setting, lay persons without therapeutic experience can be trained in delivering the intervention, simplifying dissemination processes in LMICs. Hence, the aim of the current study was to evaluate Pennebaker's unstructured writing paradigm in an RCT regarding long-term consequences of early parental loss assessed as grief, PG, and depressive symptoms in orphaned Rwandan adolescents.

## Method

### Participants

A sample of orphans in rural Rwanda (Rwamagana district) was recruited in early 2009. The adolescents were living in an orphanage or visiting the orphanage's boarding school. Inclusion criteria were: 1) loss of one or both parents, 2) age between 14 and 18 years, 3) no severe intellectual impairment (as judged by the school's teachers), and 4) the participants’ and their guardians’ agreement to participate (assurance was given that participation was voluntary). Because of their age, the majority of participants may not have been able to remember the genocide, as they were between 1 and 4 years old at the time of the incident. However, as mentioned in the introduction, all of them were well aware of the genocide and their parents’ deaths.

### Procedure

**Fig. 1:**
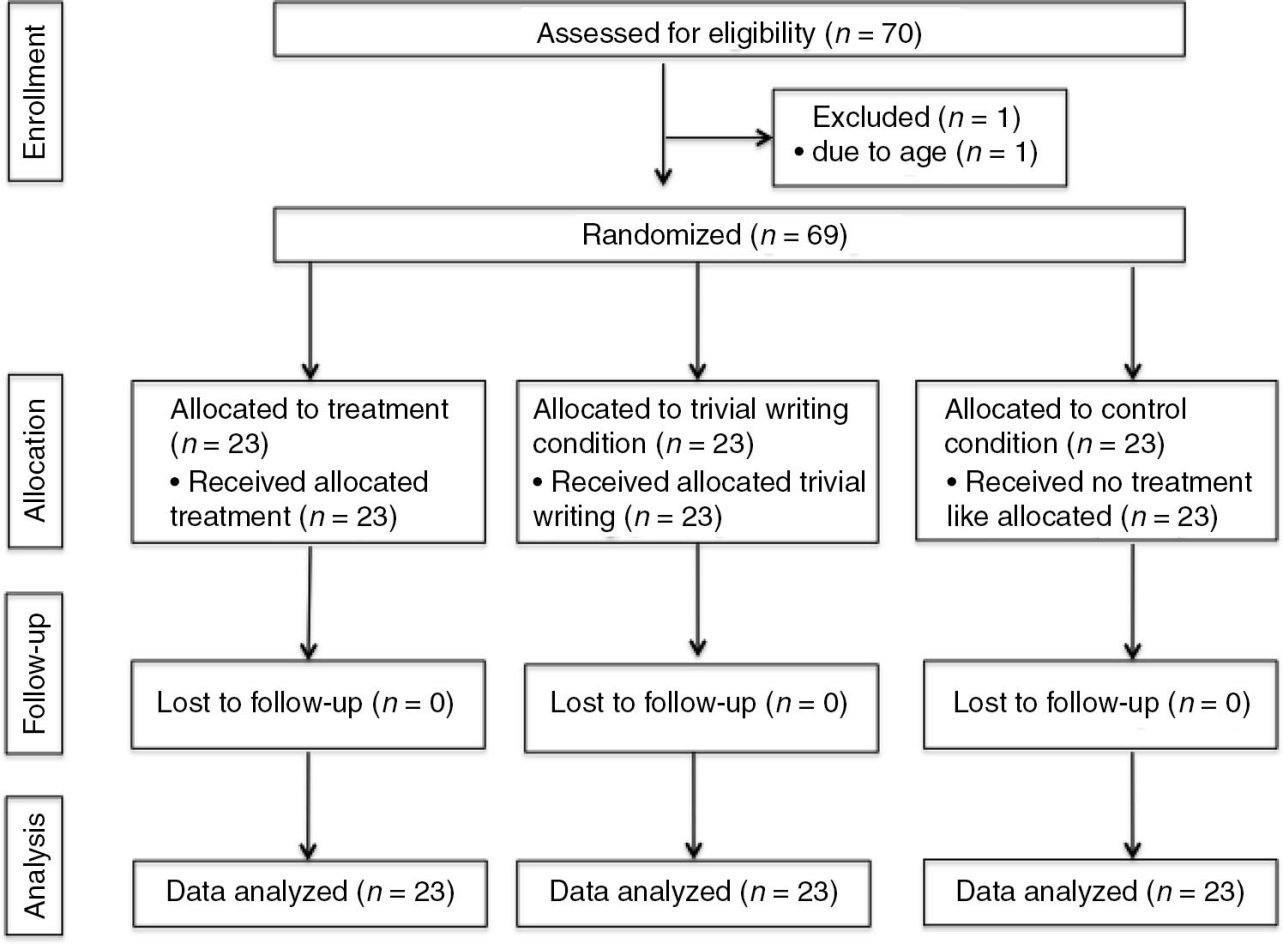
Participant flow.

The school's and the orphanage's management invited all orphans in the relevant age group to an informative meeting which was presented by the first author and translated into Kinyarwanda. The adolescents (*N*=70) were encouraged to ask questions at any time. All individuals invited were willing to participate and their guardians gave written consent; one had to be excluded due to his age (13 years). At baseline (T1) and at posttest (T2), the subjects completed self-report questionnaires. The individuals were assigned to one of three experimental conditions using stratified randomization with regard to age and sex. This approach was chosen to ensure balance of these characteristics in each group despite the small sample size (Moher et al., 2010). The responsibilities of the investigator (J.U.) included inviting the adolescents to assessments, ensuring that all questionnaires were completed, and coordinating the writing sessions. Thus, she was not blind to the experimental conditions. The flow of participants is illustrated in Fig. 1. The study was reviewed by the ethical board of the Ludwig-Maximilians-University, Munich.

### Experimental conditions

All instructions were translated and back-translated, given in written form, and all adolescents wrote in their mother tongue Kinyarwanda. The writing conditions followed Pennebaker's manual, *Hints on running a writing experiment* (Pennebaker, 1994). During the study, adolescents were able to meet with their guidance counselor whenever they felt stressed, sad, or depressed. After a break of 1 h after classes, 30-min writing periods started at 5 p.m. each week on three consecutive Thursdays. Baseline assessment took place 1 week before writing started and the postassessment 1 week after writing finished. Both assessments and the writing intervention were conducted in the school's assembly hall. The investigator was present the whole time, gave instructions at the beginning, and answered questions where needed. The instructions remained the same every week and indicated that the participants could write about the same things every week if they wished, but continuous writing during the time of the writing session was important. Anonymity was ensured by providing a wooden box with a small slit locked with a padlock to place the essays inside. No names were written on any of the papers.

Emotional Writing (EW, condition 1): The EW participants were asked to write about their deepest emotions concerning their loss. The writing instructions were adapted for the loss of an attachment figure:

(…) In the next 30 minutes, I want you to write about your deepest emotions and thoughts regarding your deceased loved one. Really let go and explore your feelings and thoughts about it. In your writing, you might tie this experience (i.e. the death of that person) to your childhood, your relationships with others, people you have loved or love now, or even school. How is this experience related to the person you would like to become, who you have been in the past, or who you are now?

Regardless of what you choose to write about, it is important that you really let go and explore your very deepest emotions and thoughts (…)

Positive Writing (PW, condition 2): In the PW condition we asked the participants to write about their favorite hobby, which is basically trivial but could also be experienced as positive topic and activating resources. Instructions were similar to those in the EW condition but focused on the importance of the hobby and its influence on their lives.

Non-writing control group (NW, condition 3): NW participants served as controls and received no further treatment within the study. These students were offered EW by one of the local educators after the conclusion of the study.

## Measures

All measures were translated and back-translated by two independent translators. Subsequently, discrepancies between the translations were discussed until agreement was reached. The measures used are disorder-specific; however, it is possible that our measures did not only assess grief or affective symptoms, but general long-term distress due to early parental loss.

### Prolonged Grief Questionnaire for Adolescents

Based on the Extended Grief Inventory (Layne, Saviak, Saltzman, & Pynoos, 2001), we developed a 36-item self-report grief measure for children and adolescents, the Prolonged Grief Questionnaire for Adolescents (PGQ-A; Unterhitzenberger & Rosner, in press). Additional items covered PG according to the criteria of Cohen et al. (2006; childhood traumatic grief). The instructions asked the participants to name their most significant loss, then: “The following statements are about how you feel about the named person's death. While answering each question please think about that person.” The measure uses a five-point Likert scale estimating the frequency of thoughts and feelings from (0) never to (4) always. The severity score ranges from 0 to 144. In addition, two questions ask about previous disclosure of the loss and impairment due to the symptoms. In the preliminary evaluation with the same sample, the PGQ-A showed good psychometric properties (Cronbach's alpha 0.94) and a cut-off of 82.5 for elevated grief resp. PG (Unterhitzenberger & Rosner, in press).

### Mini International Neuropsychiatric Interview for Children and Adolescents, Part A

To assess depressive symptoms, we used the nine-item Mini International Neuropsychiatric Interview for Children and Adolescents, Part A (MINI-KID A; Sheehan et al., 2010), which provides a major depression diagnosis according to DSM-IV. We included a five-point Likert scale, (0) never to (4) always, during the last four weeks for each item so it could be used as a self-report form, which allowed for a maximum score of 36. The reliability for the Kinyarwandan version was satisfying (Unterhitzenberger & Rosner, in press). To define elevated symptoms we chose a cut-off ≥20, as at least five items have to be met almost daily when used as an interview.

### Data analysis

To test for group differences on the sociodemographic information and the baseline measures, we used contingency tables with chi-squared tests for frequency variables and analyses of variance (ANOVA) for continuous variables. We computed sum scores for each participant at pre- and posttest on PGQ-A and MINI-KID A. Paired samples *t*-tests were used to test for pre–post changes within the three experimental conditions. To examine differences between groups over time, we used a repeated measures ANOVA with PGQ-A scores as dependent variable and experimental condition as independent variable. We did the same analysis with MINI-KID A scores. In case of significance—set at ≤0.05—post hoc *t*-tests with Bonferroni-Holm adjustment were conducted. Accordingly, statistical significance levels were set at *p*<0.017 and <0.025. Non-significant results are marked *n s*. To test for differences in treatment outcome between students with elevated versus low grief, we conducted a repeated measures ANOVA with PGQ-A scores as dependent variable, and intervention condition and grief severity as independent variables. Eta-squared (*η* ^2^) is given as a measure of strength.

## Results

Demographic characteristics for each experimental condition are presented in Table 1. No significant differences between the conditions on any demographic variable were found; hence data are not provided. The 69 participants were between 14 and 18 years old (*M*=16.30, SD=1.17), and 33 were female (47.8%). Approximately half of the participants were living in the orphanage (50.7%). In total, 35 youths (50.7%) had lost both parents. The majority of the participants had their most significant loss due to murder. The average time that had passed since the loss was *M=*13.14 years (SD=2.30). Most participants had not disclosed their grief previously by seeking help in conversations with professionals (e.g., teachers, doctors, or priests) which underlines that these adolescents do not receive the support they need.

**Table 1:** Background, loss characteristics, and psychopathology

	EW (*n=*23)	PW (*n=*23)	NW (*n=*23)
Variable	*M* (SD)	*M* (SD)	*M* (SD)
Years attending school	8.2 (1.9)	8.0 (1.8)	7.9 (2.2)
Time since loss	13.2 (1.9)	12.8 (2.9)	13.4 (2.0)
	*n* (*%*)	*n* (*%*)	*n* (*%*)
Residence			
Relatives	14 (60.9)	11 (47.8)	9 (39.1)
Orphanage	9 (39.1)	12 (52.2)	14 (60.9)
Deceased			
Mother	2 (8.7)	1 (4.3)	1 (4.3)
Father	7 (30.4)	12 (52.2)	10 (45.0)
Both	14 (60.9)	10 (43.5)	12 (50.7)
Cause of death			
Murder	18 (78.3)	17 (73.9)	16 (69.6)
Accident	0	0	2 (8.7)
Illness	5 (21.7)	5 (21.7)	4 (17.4)
Other	0	1 (4.3)	1 (4.3)
Disclosure of grief before			
Yes	11 (47.8)	6 (26.1)	6 (26.1)
No	12 (52.2)	17 (73.9)	17 (73.9)
Grief severity (PGQ-A)			
Elevated (range, 82,5–144)	16 (69.6)	9 (39.1)	9 (39.1)
Depression severity (MINI-KID A)			
Elevated (range, 20–36)	12 (52.2)	7 (30.4)	5 (21.7)

EW=emotional writing, PW=positive writing, and NW=non-writing; PGQ-A=Prolonged Grief Questionnaire for Adolescents; MINI-KID A=Mini International Neuropsychiatric Interview for Children and Adolescents, Part A.

The participants in the three groups differed regarding their baseline average sum scores on the PGQ-A, *F*(1, 69)=3.317, *p*=0.042. The T1- and T2-scores as well as statistical tests are shown in Table 2. In EW, 69.6% (*n*=16) scored above the cut-off for clinically relevant PG, whereas this was true for 39.1% (*n*=9) in PW and NW, respectively (Table 1). Overall, 49.9% (*n*=34) scored above the cut-off. There were no significant differences in MINI-KID A scores between groups at baseline, *F*(1, 69)*=*1.258, *n s*. Mean symptom scores were moderate to high, with elevated scores in 52.2% (*n*=12) of EW, 30.4% (*n*=7) of PW, and 21.7% (*n*=5) of NW, respectively. Overall, 34.8% (*n*=24) of participants had high scores. Ten adolescents (14.5%) scored above the cut-off on both PGQ-A and MINI-KID A.

**Table 2:** Means, standard deviations, and statistical tests for the PGQ-A and MINI-KID A at the pre- and posttest stages

	EW (*n*=23)			PW (*n*=23)			NW (*n*=23)			*F* between (*p*)	
	
	T1 *M* (SD)	T2 *M* (SD)	*t* (*p*)	T1 *M* (SD)	T2 *M* (SD)	*t* (*p*)	T1 *M* (SD)	T2 *M* (SD)	*t* (*p*)	T1	T2
PGQ-A	92.1 (32.6)	87.6 (32.6)	1.519 (0.143)	73.0 (31.8)	63.9 (34.9)	2.189 (0.039)	69.9 (30.4)	60.3 (30.1)	2.143 (0.043)	3.317 (0.042)	4.753 (0.012)
MINI-KID A	18.8 (10.6)	20.6 (11.4)	−1.292 (0.210)	14.0 (10.6)	13.0 (10.0)	0.977 (0.339)	15.7 (9.9)	12.1 (10.3)	2.529 (0.019)	1.258 (0.291)	4.474 (0.015)

EW=emotional writing, PW=positive writing, and NW=non-writing; PGQ-A=Prolonged Grief Questionnaire for Adolescents; MINI-KID A=Mini International Neuropsychiatric Interview for Children and Adolescents, Part A.

There were no significant changes in grief symptoms from pre to post in the EW group, *t*(22)=1.519, *n s*. Significant decreases over time were found for PW, *t*(22)=2.189, *p=*0.039, and NW, *t*(22)=2.143, *p=*0.043.

**Fig. 2:**
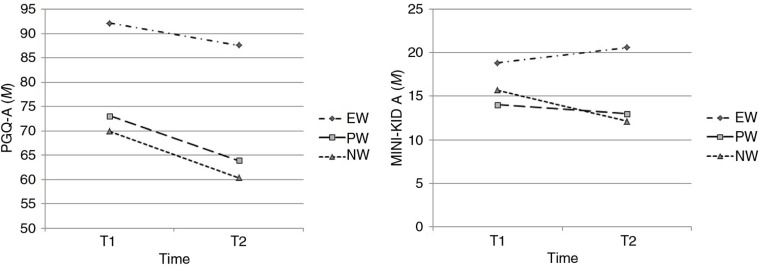
Changes from pre- to posttest in grief (PGQ-A) and depression (MINI-KID A) severity across the three experimental conditions.

The interaction in the 3 (Condition)×2 (Time) ANOVA was not significant, *F*(2, 69)=0.518, *n s* (Fig. 2). Therefore, no differential effect regarding the intervention condition was found. However, there was a significant main effect of time, *F*(1, 69)=11.671, *p*=0.001, *η* ^2^=0.150.

In a further analysis, we compared participants with elevated (*n*=34) and low grief severity (*n*=35). The 2 (Grief Severity)×2 (Time) ANOVA resulted in a significant interaction, *F*(1, 69)=4.630, *p*=0.035, *η*=0.065, indicating significantly higher changes from pre- to posttest for the group with higher PG. Regarding experimental conditions, a repeated measures 2×3×2 (Time×Intervention×Grief severity) ANOVA showed no significant effect for the interaction, *F*(2, 69)=1.346, *n s*, indicating that higher scores generally predicted the reduction of grief symptoms independent of the experimental conditions.

Subjects in the EW showed no significant change in depression scores at T2, *t*(22)*=*−1.292, *n s* (Fig. 2). In a between-subject analysis (within the EW), in which those who showed improvements were compared to those whose condition worsened, no significant differences in other variables (e.g., sex, age, number of losses, depression severity at baseline) were found; hence, data are not provided. Symptoms in the PW did not change, *t*(22)=0.977, *n s*, whereas symptom severity declined significantly in the NW, *t*(22)=2.529, *p=*0.019. The repeated measures 2×3 (Time×Intervention) ANOVA using MINI-KID A scores showed a significant interaction, *F*(2, 69)=4.267, *p*=0.018, *η* ^2^=0.114. This interaction reflected that participants in the NW, *t*(44)=2.948, *p*=0.005, and in the PW, *t*(44)=2.380, *p*=0.022, each showed significantly higher improvements than the EW.

## Discussion

This study contributes to the literature on grief interventions for children in LMICs which shows a scarcity of effective treatments and evidence-based evaluations. Our RCT tested school-based unstructured writing on grief and depression in a country that had been struck by a civil war which left many children orphaned. Even though the losses dated back an average of 13 years, our adolescents reported high symptom levels. Therefore, psychosocial help was clearly needed. Our results do not support unstructured writing for grieving adolescents.

We cannot answer the question comprehensively whether the loss-related distress reported in our sample really reflects PG as seen in individuals who remember the lost person and the actual circumstances of the loss in all instances. Yet, our findings are in accordance with previous studies reporting on rates of traumatic stress and PG in Rwanda more than 10 years after the genocide (Mutabaruka, Séjourné, Bui, Birmes, & Chabrol, 2012; Schaal et al., 2010). Our high rates might inter alia be due to our kind of assessment: Self-report is assumed to produce higher agreement than interviews. Furthermore, it is possible that the adolescents might have reported their level of overall distress rather than really rating each item individually.

We have to reject the hypothesis that unstructured writing is effective in reducing PG or depressive symptoms in long-term grieving adolescents. The first possible reason for this result is that our intervention provided little structure, little elaboration, and no feedback, in contrast to more effective writing interventions like WfR (Kalantari et al., 2012). Research on the Pennebaker (1997) paradigm has made a lot of progress, and organizations must acknowledge that simple writing can be useful for stressed but healthy students but is not indicated for orphans affected by long-term loss. Second, the postassessment took place a short time after the completion of the writing sessions (Bower et al., 2003; Sloan & Marx, 2004). Although WfR showed significant intervention effects 1 week after writing (Kalantari et al., 2012), Wittouck and colleagues (Wittouck, Van Autreve, De Jaegere, Portzky, & Van Heeringen, 2011) hypothesized the aim of PG work as reaching the level of normal grief, which is in part processed subsequently. As we did not collect follow-up data, we are unable to contribute to this discussion. However, only two out of 12 studies in the review of mental health interventions for young people affected by war report follow-up data at 9 or 12 months (Jordans et al., 2009). Third, as many years have passed since our participants sustained their losses, it is possible that they did not have enough memories of their parents or their parents’ deaths. On the other hand, the lack of memory could also be the content of the essays. As no content analysis was done, we cannot judge the influence of this factor on our results. In addition, it is possible that EW was not indicated in our sample: Writing about the deepest emotions might have stirred up negative emotions. Studies on writing and grief have shown best results in both adults and adolescents when specific strategies for grief processing were offered (Lichtenthal & Cruess, 2010; Wagner et al., 2006; Kalantari et al., 2012). Also Stroebe and colleagues summarize that ruminating over a loss is supported by the absence of instructions that focus on processing the grief (Stroebe & Stroebe, 1991; Stroebe et al., 2002). Last, in line with other studies (Houwen et al., 2010; Segal, Bogaards, Becker, & Chatman, 1999; Stroebe et al., 2002), Kovac and Range (2002) concluded that writing therapy may be an ineffective approach for the treatment of affective disorders. We cannot provide any evidence pertaining to that assumption with our data.

Unexpectedly, we found significant improvements in both control groups. Adolescents writing positively showed a significant reduction in PGQ-A scores 1 week after writing was completed. Effects of positive mood induction are not expected to last this long. Similar to our results there are a number of studies reporting no significant differences between disclosure and PW (Burton & King, 2004; King, 2001; Lewandowski, 2009; these studies were not examining grief reactions). It is possible that writing about a positive activity helped shift adolescents’ focus on the positive aspects of their lives. However, this argument does not fit for the NW group. In the MINI-KID A, NW participants showed best results, whereas scores in EW slightly increased. As time since bereavement was on average 13 years, a time effect seems unlikely. Possibly, even with the Rwandan focus on remembering and mourning the genocide, when our sample completed the self-report measures it may have been the first time the adolescents reflected on its effects on their individual lives. This may have stimulated thoughts and conversations about their losses and their grief. Also, study participation, a feeling of belonging as all participants lost someone, and a feeling of being cared about can be further reasons why the NW group improved. However, this does not explain why they improved the most. Here, a follow-up assessment would have been especially illuminating. In addition, a Solomon four group design (Solomon, 1949) could have given more information on variables that influenced our findings.

We conducted a universal intervention (i.e., no cut-off for inclusion in study) which is regularly found with school-based approaches (Persson & Rousseau, 2009) and within grief treatments for children (Currier, Holland, & Neimeyer, 2007; Rosner, Kruse, & Hagl, 2010). The necessary examination of the effects for participants with elevated versus low grief scores showed that individuals with elevated scores benefited more from the intervention than the others (regardless the allocated group). This might be another factor explaining the very small overall effects in our study and is in line with meta-analyses on childhood grief treatment (Currier et al., 2007; Rosner et al., 2010).

### Limitations

One major limitation is the small sample size in general and the resulting small groups in the experimental conditions. No power analysis was done. As we were unable to collect follow-up data, statements on long-term effects are not possible. Furthermore, we did not recruit a representative sample but present findings from a convenience sample. We randomized all adolescents who were assessed, which led to a high variance between the subjects. Grief scores differed significantly among the three conditions before treatment and randomization was stratified.

Time since bereavement was long for all participants. Thus it is open to discussion whether 1) grief as main outcome, and 2) EW as experimental condition were well chosen here, as adolescents had probably only few memories of their murdered parents. As essays were not analyzed by content and no feedback was given, we do not know whether participants wrote according to the instructions.

## Conclusions

As there is a pronounced scarcity of evaluated treatments for adolescents in LMICs, we would like to highlight the importance of publishing non-significant or negative results “to stop wasting resources on interventions that either do no good or even do harm” (Yule, Dyregrov, Raundalen, & Smith, 2013, p. 4). To sum up, the following lessons can be learned from the above:

1. Intervention studies based on young people in LMICs are needed to support evidence-based practices under the respective environmental conditions.
2. When evaluating a grief intervention, researchers should focus on indicated instead of universal interventions.
3. In order to explore if there are long-term or delayed effects, follow-up data are needed.
4. Concerning writing interventions, individualized interventions are possibly superior to simple writing interventions.
5. Participating in a grief study may have benevolent consequences even without an intervention; in future research, a Solomon four group design (Solomon, 1949; two treatment and two control groups, one of each group is not pretested) is recommended to evaluate the effects of participating in a grief study and to control for possible positive effects of grief questionnaires.

## Author Note

This article was part of the first author's doctoral dissertation, which was submitted at Ludwig-Maximilians-University Munich. Parts of the article were presented at the ISTSS 2012 in Los Angeles. We thank all participating adolescents and caregivers in Rwanda for their great trust and support.
